# Mesenchymal Stem Cell-derived Exosomes: Novel Therapeutic Approach for Inflammatory Bowel Diseases

**DOI:** 10.1155/2023/4245704

**Published:** 2023-04-04

**Authors:** Cheng-mei Tian, Mei-feng Yang, Hao-ming Xu, Min-zheng Zhu, Yuan Zhang, Jun Yao, Li-sheng Wang, Yu-jie Liang, De-feng Li

**Affiliations:** ^1^Department of Gastroenterology, Shenzhen People's Hospital (The Second Clinical Medical College, Jinan University, The First Affiliated Hospital, Southern University of Science and Technology), Shenzhen, Guangdong, China; ^2^Department of Emergency, Shenzhen People's Hospital (The Second Clinical Medical College, Jinan University, the First Affiliated Hospital, Southern University of Science and Technology), Shenzhen, 518020 Guangdong, China; ^3^Department of Hematology, Yantian District People's Hospital, Shenzhen, Guangdong, China; ^4^Department of Gastroenterology and Hepatology, Guangzhou Digestive Disease Center, Guangzhou First People's Hospital, School of Medicine, South China University of Technology, Guangzhou, China; ^5^Department of Medical Administration, Huizhou Institute of Occupational Diseases Control and Prevention, Huizhou, Guangdong, China; ^6^Department of Child and Adolescent Psychiatry, Shenzhen Kangning Hospital, Shenzhen, Guangdong, China

## Abstract

As double membrane-encapsulated nanovesicles (30-150 nm), exosomes (Exos) shuttle between different cells to mediate intercellular communication and transport active cargoes of paracrine factors. The anti-inflammatory and immunomodulatory activities of mesenchymal stem cell (MSC)-derived Exos (MSC-Exos) provide a rationale for novel cell-free therapies for inflammatory bowel disease (IBD). Growing evidence has shown that MSC-Exos can be a potential candidate for treating IBD. In the present review, we summarized the most critical advances in the properties of MSC-Exos, provided the research progress of MSC-Exos in treating IBD, and discussed the molecular mechanisms underlying these effects. Collectively, MSC-Exos had great potential for cell-free therapy in IBD. However, further studies are required to understand the full dimensions of the complex Exo system and how to optimize its effects.

## 1. Introduction

Inflammatory bowel disease (IBD) is a chronic recurrent intestinal inflammation mediated by abnormal immunity, and its pathogenesis is involved in genetic susceptibility, physicochemical environment, intestinal microecology, oxidative stress, and other factors. The incidence and morbidity of IBD have been gradually increasing year by year [1–6].

Currently, the conventional drugs for IBD include aminosalicylic acid preparations, glucocorticoids, and immunosuppressive agents. However, the current treatment of IBD faces several challenges, such as fewer drug choices, large dosages, and extended maintenance time, leading to many serious side effects, while there is still a high rate of ineffective treatment or drug dependence [7]. In addition, the rates of surgical intervention and colon cancer transformation remain high [8]. Therefore, it is urgently necessary to develop alternative treatment options with fewer side effects.

Biologics is an alternative [9]. Treatment with infliximab (IFX) may be considered for patients who are refractory to conventional drugs or who are drug dependent [10]. IFX regulates the abnormal immunity of IBD patients with tumor necrosis factor-*α* (TNF-*α*) as a therapeutic target, and domestic and foreign studies have confirmed its efficacy in treating IBD [11–13]. However, the therapeutic target of IFX is single, and subsequent clinical studies have found that about 50% of patients have primary or secondary failure to respond to IFX therapy [14–16]. Currently, most other biological agents are still in the initial research stage, and the mechanism of action (MoA) and clinical response need to be further studied [9].

Mesenchymal stem cells (MSCs) belong to adult pluripotent stem cells [17], widely distributed in the human body, mainly in tissue stroma, such as bone marrow (BM), umbilical cord, placenta, amniotic fluid, and fat [18]. MSCs have the ability of active proliferation, multidirectional differentiation potential, as well as strong immunomodulatory capacity [19]. Animal studies have shown that transplantation of MSCs affects intestinal repair and immune regulation in IBD [20, 21], which is expected to become an emerging treatment for IBD [22]. Unfortunately, few MSCs (< 1%) are eventually recruited to the site of intestinal inflammation after transplantation [23–26]. However, infusion of MSC-derived exosomes (MSC-Exos) can produce the same efficacy as MSC transplantation [27–31]. Moreover, MSCs regulate the biological activity of target cells mainly through paracrine-mediated substance transport or signal transduction. As the most critical paracrine carrier, Exos can play most of the roles of MSCs [30–32]. In this review, we summarized the most recent advances in the properties of MSC-Exos, provided a comprehensive review of MSC-Exos in treating IBD, and discussed the molecular mechanisms underlying these effects. Finally, challenges and future directions in this field were presented to advance the development of IBD therapy.

## 2. Biological Properties of MSC-Exos

### 2.1. Basic Features of MSC-Exos

**Figure 1:**
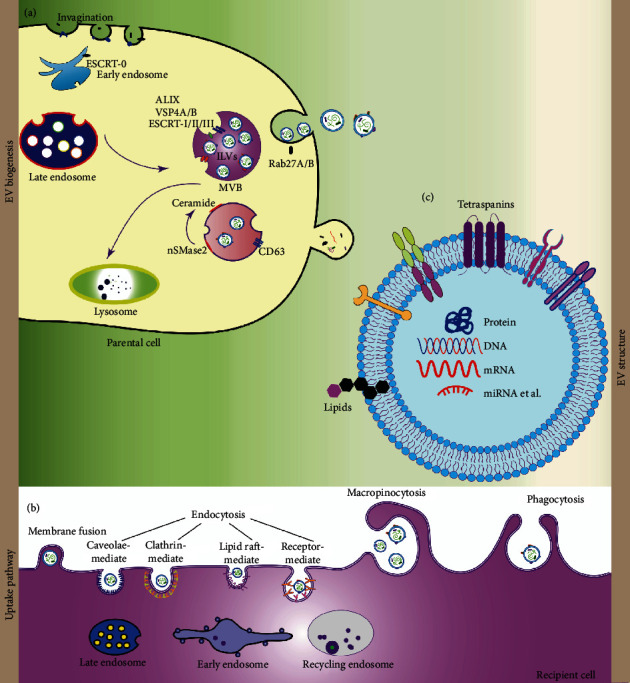
Biogenesis, composition, and uptake pathway of exosomes. (a) Exosomes were derived from the endosomal pathway. Extracellular biomaterial enters the cytoplasm through endocytosis pathway, then fuses with early endosome and develops into late endosome. Then, forms intraluminal vesicles (ILVs) containing vesicle structure in MVBs. When MVB fuses with lysosomes, it degrades the contents. When MVBs were transferred to plasma membrane resulting in the release of exosomes into the extracellular space. Endosomal sorting complex (ESCRT)-independent and dependent pathways required for exosome transport are involved in various stages of exosome formation. (b) Exosome components include proteins, lipids, nucleic acids, and small molecules, as well as surface proteins. (c) Exosomes can exert their effects by binding to receptors present on the surface of target cells, or through endocytosis, membrane fusion, macrophage.

As particles naturally released from cells wrapped by a lipid bilayer membrane, extracellular vesicles (EVs) have a diameter of about 30-3,000 nm and cannot replicate themselves [31, 33–35]. According to the vesicle size, EVs can be artificially divided into "small extracellular vesicles (sEVs)" with a diameter of less than 200 nm and “medium and large extracellular vesicles (m/lEVs)” with a diameter of more than 200 nm [36]. Among them, Exos are sEVs with a diameter of about 30-150 nm and a density of 1.10-1.18 g/mL, the main form of paracellular secretion [37]. Exos originate from intranuclear bodies. Many primary endocytic vesicles containing maternal cell components, which originate from the inversion of the cell plasma membrane, fuse with each other to form early endosomes after the strict screening and then become mature into late endosomes after the screening. Only a few late endosomes can encapsulate the intraluminal vesicles to form multivesicular bodies, which are then released into Exos by budding under the regulation of RAB, a member of the GTPase family. However, more endosomes fuse with lysosomes, degrading their contents (Figure 1) [34, 38–47].

MSC-Exos can mediate the material exchange and signal communication between cells [30, 31, 48]. MSC-Exos can recognize specific receptor molecules loaded into the environment by using the specific markers on the membrane surface as ligands and can also be recognized and bound by the specific receptors on the surface of the target cell membrane. The combination with the target cell can directly activate the signal transduction pathway in the target cell. It can further act on the target cell through membrane fusion or endocytosis to deliver bioactive substances and activate the signal transduction pathway in the target cells [49, 50]. The fusion of Exos with the target cell membrane and the release of bioactive substances, such as miRNA, mRNA, and protein, into the target cell, further affect the biological behavior of the target cells [51, 52].

Compared with cell therapy of MSCs, MSC-Exos have lower immunogenicity [53, 54], which can avoid the potential risks of cell therapy, such as immune rejection [35, 54–56], risk of tumorigenesis [57–59], coagulation abnormalities [60–65], and functional decline [66, 67]. Moreover, as low-immunogenicity nanoscale carrier cysts, MSC-Exos can carry therapeutic drugs to target cells to enhance drug efficacy and reduce adverse reactions [68–71]. MSC-Exos can penetrate various physiological barriers in the human body, including the blood-brain barrier [72]. Exos can exist stably in a variety of body fluids. We can find new diagnostic biomarkers for diseases by detecting Exos in body fluids. In addition, we can study the Exos of various cells simultaneously and find new ways to treat diseases [73, 74].

### 2.2. Components of MSC-Exos

The contents of MSC-Exos are extremely rich, including protein, nucleic acid, and lipid. They are the material basis of the biological activity and function of MSC-Exos [75]. The contents of Exos are closely related to the type, source, and growth environment of mother cells. The main difference lies in the protein and nucleic acid molecules strictly screened by the mother cell [76, 77].

#### 2.2.1. Protein

According to ExoCarta (http://www.exocarta.org/), more than 1,000 kinds of proteins can be extracted and separated from MSC-Exos [78]. Most proteins exist in the Exo membrane, and their types and contents are greatly affected by the mother cell and growth environment [36]. It is believed that the proteins entering the exocrine body have been strictly screened [75]. The future of newly synthesized proteins in ribosomes depends on their amino acid sequence, which contains a particular signal sequence, also known as a signal peptide, as a sorting signal to guide the protein to the target compartment. If a protein is loaded into a vesicle, the corresponding receptor on the corresponding vesicle membrane must recognize its sorting signal. Exos from different MSCs have no difference in the expression of surface marker proteins, mainly Alix, TSG101, CD9, CD81, CD63, CRR2, and HSP70, which are helpful to be recognized and bounded to target cells [43, 79].

#### 2.2.2. Nucleic acid

MSC-Exos contain various types of nucleic acid, mainly mRNA, miRNA, and DNA. It is an important marker to distinguish from other EVs [75]. Among them, mRNA can participate in the cell cycle, angiogenesis, and histone modification. miRNA can be transported to target cells through Exos, inhibit the transcription and translation of target mRNA, or directly degrade target mRNA. Moreover, miRNA can regulate the gene expression of target cells, participate in the growth, differentiation, and apoptosis of target cells, and play a role in mediating immunity, regulating inflammation, and repairing damage [80, 81]. Similar to the intracellular transport of protein, the intracellular transport of nucleic acid should also have a corresponding recognition sequence. However, the specific mechanism of Exo screening nucleic acid molecules remains largely unclear [76, 77]. The content of nucleic acid molecules in Exos is small, and the difference is large. Even if Exos are extracted from the same cell source, culture condition, and separation method, their nucleic acid molecules are also different [81].

#### 2.2.3. Lipid

MSC-Exos have few lipid types and minor differences but rich content [75]. The lipid bilayer of Exos contains abundant phospholipids, glycolipids, and cholesterol to maintain the morphology and structure of the exocrine body [39]. Lipid components, such as cholesterol, sphingomyelin, and ceramide, can participate in the information transmission of the exocrine body as signal molecules [75]. In addition, cholesterol, oxysterol, ceramide, and lipid transporter are involved in the production of the exocrine body [82].

### 2.3. Preparation of MSC-Exos

The MSC-Exo preparation used in the study is derived from an MSC medium (solution) [37, 80]. The first is the acquisition and cultivation of MSCs. The biological function of MSCs is affected by cell source and culture conditions, while the cell purity and physical function of MSC culture affect the natural function of MSC-Exos [36]. The second is the extraction, identification, and preservation of MSC-Exos. The separation and purification of MSC-Exos usually use centrifugation, ultrafiltration, precipitation, antibody affinity capture (immunomagnetic bead method), and microfluidic chip method. There are various methods with obvious advantages and disadvantages. The most appropriate method should be selected according to the research needs [83]. The MSC-Exo preparations are defined through physical-biochemical and functional attributes, mainly to detect the purity, active ingredients, and biological functions of MSC-Exos. At present, the recognized identification method of MSC-Exo is to observe its shape and size by electron microscope, determine its surface marker by protein imprinting technology, and measure its particle size and concentration by nanoparticle tracking analysis technology [83, 84]. In order to improve the scientificity and preciseness of the study, the identification of MSC-Exo preparations should meet the minimum standard of sEVs identification recommended by 2018 ISEV, improve the consistency and repeatability of the study, but do not limit the flexibility and diversification of production technology [37].

## 3. Mechanism of MSC-Exos in the Treatment of IBD

**Figure 2:**
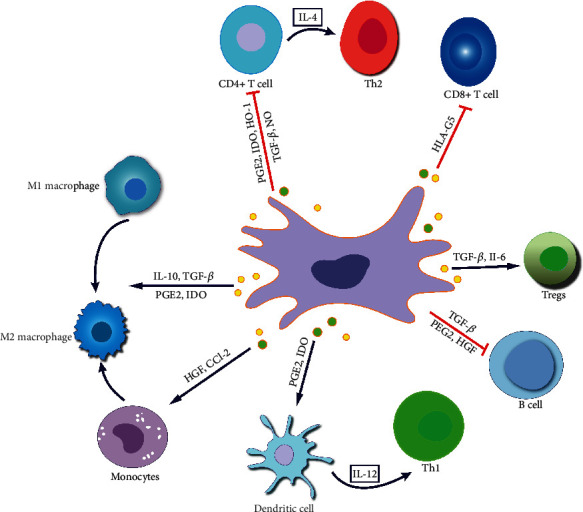
The effects of MSC-EVs on immune effector cells in IBD.

Studies have shown that MSC-Exos can target the sites of intestinal inflammation, act on immune cells, such as macrophages, T lymphocytes, and dendritic cells (DCs) and regulate the phenotype and function of immune cells by delivering bioactive substances, such as cytokines, to regulate abnormal immunity and inhibit inflammatory response (Figure 2) [51]. In addition, MSC-Exos can also act on intestinal epithelial cells (IECs), repair the intestinal epithelial barrier (IEB), inhibit oxidative stress, and suppress colon fibrosis. Therefore, it can cure IBD [85].  Table 1 shows the therapeutic application of MSC-Exos in IBD treatment.

### 3.1. Macrophages

Stimulated by pro|inflammatory factors, macrophages can be recruited to inflammatory sites and differentiate into macrophages with different polarity by chemokines and inflammatory factors. M1 macrophages secrete proinflammatory cytokines (IL-1 *β*, IL-6, TNF- *α*, and IL-12) and Th1 chemokines (CXCL9, CXCL10, and CXCL11), which play a role in antigen presentation, activating T cells and inducing an adaptive immune response. M2 macrophages secrete inhibitory cytokines such as IL-10 and TGF-*β* to downregulate immune responses and inhibit inflammatory responses (Figure 1) [86, 87]. The abnormal polarization of macrophages causes intestinal mucosal immune abnormalities and mediates intestinal inflammatory response, one of the initiating factors in the onset of IBD [88]. Studies have shown that regulating the polarization direction of macrophages, and the ratio of M1/M2 macrophages is essential in IBD immunotherapy [89–91]. Both in vitro studies and animal experiments have shown that the lipopolysaccharide (LPS)-stimulated macrophages are cocultured with BM-derived MSC-Exos. After 24 h, MSC-Exos labeled with green fluorescence are found in macrophages, which regulate the polarization of macrophages to M2 and reduce the M1/M2 ratio while downregulating the expressions of IL-6, IL-7, TNF-*α*, and IL-12 and reducing the infiltration of macrophages in colon tissue [92, 93]. Further studies have shown that adipose-derived MSC-Exos contain an anti-inflammatory factor, TNF-*α* stimulated gene-6 (TSG-6), which may be an essential protein in regulating the polarization of M2 macrophages [94]. The possible pathways of MSC-Exos regulating macrophages are as follows:

*Transfer of miRNA*: MSC-Exos binding to macrophages at the site of intestinal inflammation release the carried miRNA into macrophages, with targeted regulation of mRNA in macrophages, and regulate the polarization of M2 macrophages. Studies have shown that the Exo-miRNAs of MSCs, such as miR-146a, downregulate the expressions of TNF receptor-related factor 6 (TRAF-6) and IL-1 receptor-related kinase 1 (IRAK-1), inhibit the release of proinflammatory cytokines, and also promote the expression of anti-inflammatory factor IL-10[95].

*Delivery of anti-inflammatory proteins*: MSC-Exo is rich in multiple proteins that inhibit colitis activity, especially metallothionein-2 (MT-2), which inhibits the intestinal inflammatory response by maintaining the integrity of the intestinal barrier and inducing the M2b macrophage polarization [96].

*Induction of Toll-like receptors (TLR)*: MSC-Exos enter inside macrophages, and they probably activate the myeloid differentiation primary response gene 88 (MyD88)-dependent signaling pathway of macrophages after the recognition of TLR3 by the carried dsRNA to induce the macrophages M2 polarization, release anti-inflammatory factors, and inhibit the inflammatory response [97].

*Competitive inhibition of CC chemokine receptor 2 (CCR2)*: under the stimulation of inflammatory factors, after CCR2 expressed on the surface of monocytes binds to CC chemokine ligand 2 expressed on the surface of macrophages, monocytes are triggered to migrate to inflammatory sites and transformed into macrophages, thus promoting inflammatory response and aggravating tissue damage. MSC-Exos highly express CCR2, which competitively binds to chemokine ligand 2 on the surface of macrophages to inhibit the recruitment and activation of monocytes, prevent the M1 macrophage polarization, and decrease the expressions of proinflammatory cytokines, such as IL-1 *β*, IL-6, and TNF-*α*, thereby inhibiting inflammatory responses [98].

*Regulation of inflammatory cytokines releases*: first, MSC-Exos extracted from MSCs induced by some inflammatory factors carry a large number of anti-inflammatory cytokines and chemokines, which can be secreted into the surrounding environment after integration by macrophages. Second, the previously elucidated multiple pathways regulate or induce the M2 macrophage polarization, upregulate anti-inflammatory cytokines, and downregulate proinflammatory cytokines to maintain the balance of anti-inflammatory factors at inflammatory sites and inhibit excessive inflammatory responses [99].

### 3.2. T Lymphocytes

Due to the constant presentation of antigens to CD4+ T cells by immune cells in the intestinal mucosa, primitive CD4+ T cells (Th0) are induced to differentiate into helper T cells (mainly subtypes such as Th1, Th2, and Th17) and regulatory T (Treg) cells under the stimulation of antigen presentation and the regulation of cytokines (Figure 2). Their differentiation direction is vital in maintaining intestinal immune balance and regulating intestinal inflammatory responses [100]. Studies have shown that the coculture of T lymphocytes with MSC-Exos downregulates cyclinD-2 and upregulates P27KIP-1, which prevents T lymphocytes from entering the S phase and inhibiting the growth and proliferation of T lymphocytes, indicating that MSC-Exos can regulate the proliferation and differentiation of T lymphocytes. MSC-Exos mainly regulate the transformation balance of Th1 and Th2 as well as Treg and Th17[101, 102].

#### 3.2.1. The Regulation of the Transformation Balance between Th1 and Th2

Th1 and Th2 cells are the two main subtypes of Th0 differentiation. Th1 cells mainly express IFN-*γ* and IL-12, while Th2 cells mainly express IL-4[103, 104]. The imbalance of Th1/Th2 subsets is closely related to the chronic inflammation of IBD [105]. The differentiation direction mainly depends on the concentration of IL-12 in the environment and the activation state of antigen-presenting cells. By regulating DCs and macrophages, MSC-Exos induce the M2 macrophage polarization, inhibit antigen presentation of DCs, and reduce the release of proinflammatory cytokines, such as IL-12, which is beneficial to the transformation of T cells into Th2 cells. Studies have proved that MSC-Exos can significantly reduce the expression of IL-12, inhibit the differentiation and proliferation of Th1 cells, and promote the transformation of T cells into Th2 cells after coculturing with T cells activated by phytohemagglutinin [106, 107]. It has been found that the TSG-6 detected by human umbilical cord MSC-Exo (hUC-MSC-Exo) regulates the immune response of Th2 and Th17 cells in mesenteric lymph nodes (MLN), downregulates proinflammatory cytokines in colon tissue, and upregulates anti-inflammatory cytokines, thus protecting intestinal barrier function [23].

#### 3.2.2. The Regulation of the Transformation Balance between Treg and Th17

Treg cells inhibit the transformation of effector T cells and intestinal adaptive mucosal immunity, reducing immune-related tissue damage in IBD. A study has shown that MSC-Exos induce T lymphocyte apoptosis and regulate the differentiation and proliferation of Treg cells by programmed death ligand and galactosin-1[108]. Treg cells express anti-inflammatory cytokines, such as IL-10 and TGF-*β*, to achieve immunosuppressive effects [97, 109, 110]. On the contrary, Th17 cells express proinflammatory cytokines to promote the inflammatory activity of IBD. Eastaff-Leung et al. have found that the ratio of Th17/Treg in the peripheral blood of IBD patients is significantly increased [111]. Moreover, Chen et al. have discovered that the ratio of Th17/Treg cells is significantly increased in mesenteric lymphoid tissue of colitis rats. In contrast, the ratio of Th17/Treg cells is dramatically decreased, markedly ameliorating colitis after injecting with MSC-Exos via the caudal vein [112, 113]. Heidari et al. have found that adipose-MSC-Exos (AD-MSC-Exos) can restore the proportion of Treg cells in the spleen of the IBD mouse model to the baseline level similar to that of normal mice and also improve the inflammation of dextran sulfate sodium (DSS)-induced colitis [114].

### 3.3. DCs

DCs are the primary antigen-presenting cells in the intestinal mucosa, which express and secrete proinflammatory cytokines, such as IL-6 and IL-12, and participate in the inflammatory response in IBD (Figure 2) [115]. Additionally, they destroy the intestinal mucosal barrier and participate in IBD tissue damage by producing reactive oxygen species (ROS) [116]. The possible mechanisms of MSC-Exos regulating DCs include the following: (1) MSC-Exo-treated DCs result in downregulation of IL-4 and IL-12 and upregulation of TGF-*β* inhibit the maturation and differentiation of DCs [107, 117, 118], induce the differentiation of T cells, affect the balance of Th1/Th2 transformation [119], and inhibit the intestinal inflammatory response [120, 121]. In addition, (2) MSC-Exos also inhibit the differentiation and maturation of DCs by regulating the TLR-NF-*κ*B signaling pathway.

In conclusion, MSC-Exos have a powerful immunomodulatory effects [122]. In the treatment of IBD, MSC-Exos carrying immunosuppressive factors affect the M2 macrophage polarization, inhibit the proliferation of Th1 and Th17, promote the differentiation of Treg cells, and induce antigen-presenting cells [123].

### 3.4. IECs

#### 3.4.1. Repair of the IEB

**Figure 3:**
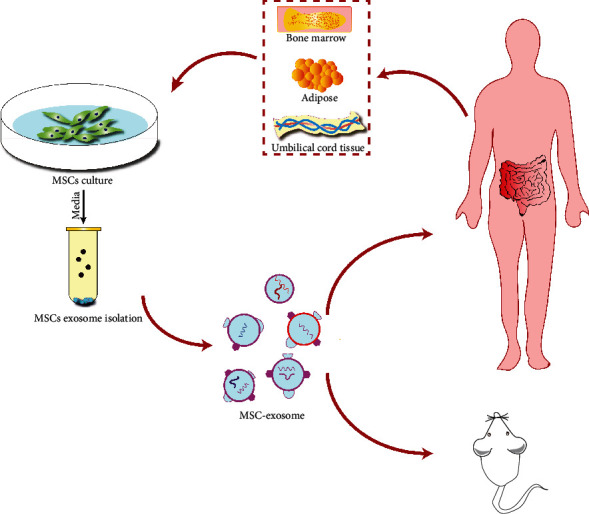
Schematic representation of IBD treatment using MSC-derived exosomes.

The intestinal barrier includes the IEB, mucosal innate immune system, intestinal mucus, and intestinal microbiota. The IEB is the tight junction of IECs, forming a complete mechanical barrier, which is the most critical part of the intestinal barrier. The imbalance and disorder in several aspects, such as mucosal immunity, surface mucus, intestinal microbiota, and oxidative stress, destroy the IEB, resulting in necrosis and apoptosis of IECs and an increase in wall permeability, which is the cause of pathological changes in IBD [124–127]. MSC-Exos can repair the injury of IECs, inhibit the apoptosis of IECs, maintain the balance of oxidative stress, and reduce intestinal wall permeability to repair the IEB (Figure 3).

*(1) Repair the Injury of IECs*. Mao et al. have found that at 12 h after intravenous injection of hUC-MSC-Exos, the labeled Exos homing to the colon tissue of colitis mice can significantly reduce the IEC damage by immune regulation [93]. Moreover, Wang et al. have found that adipocyte-derived stem cells-Exos (ADSCs-Exos) treated with vascular endothelial growth factor C (VEGF-C) promote proliferation, migration, and lymphangiogenesis of lymphatic endothelial cells (LECs) by upregulating miR-132 and regulating target cell Smad-7 gene and TGF-*β*/Smad signaling pathways [128]. In addition, a study has shown that in the DSS-induced colitis model, hUC-MSC-Exos inhibit neddylation through miR-326 to regulate the balance between ubiquitination and deubiquitination of functional proteins, promote protein degradation, or maintain protein biological activity, thus repairing IEC damage and maintaining IEB integrity [67]. However, the specific regulatory process and molecular mechanism remain unclear.

*(2) Inhibition of the Apoptosis of IECs*. He et al. have found many apoptotic bodies in the colon mucosa of ulcerative colitis (UC) patients [125]. Subsequent studies have found that IECs lack NF-*κ*B, and reduction of NF-*κ*B is the critical factor leading IECs to enter the apoptosis program [124]. In 2015, Yang et al. have found that the colon tissue of rats with colitis highly expresses caspase 3, caspase 8, and caspase 9 in the cysteinyl aspartate-specific protein (caspase) family. After injection of MSC-EVs into the tail vein, the expressions of caspase 3, caspase 8, and caspase 9 in the colon tissue of rats with colitis are increased. Conversely, the expressions of caspase 9, caspase 8, and caspase 3 in colon tissue are significantly decreased [129]. In 2019, Jang et al. have also obtained the same results. This finding indicates that MSC-EVs can inhibit the apoptosis of IECs and repair the IEB [130].

*(3) Inhibition of Oxidative Stress*. Oxidative stress is involved in the occurrence and development of IBD. The oxidation and antioxidation of colonic tissue cells are in a dynamic balance. However, chronic colonic inflammation leads to excessive production of ROS by IECs and macrophages, resulting in colonic tissue damage [131, 132]. It has been found that a significant increase of ROS is detected in the colon mucosa of IBD patients and animal models, which is positively correlated with the disease severity and the elevation of peroxidation products, such as peroxidated lipid molecules and peroxidated modified proteins [133–139]. Overproduced ROS directly damages nucleic acid, lipid, and protein molecules in colon epithelial cells through oxidation, induces necrosis or apoptosis of IECs, and destroys the IEB integrity [140]. On the other hand, oxidative stress helps the immune system clear pathogens, while excessive oxidative stress can stimulate macrophages to produce proinflammatory cytokines and aggravate the intestinal inflammatory response [141–143].

Studies have shown that MSC-Exos can repair various injuries caused by oxidative stress [144]. However, most focus on the repair effect of an oxidative stress injury in tissues, such as the heart, lungs, liver, and kidney. There are very few studies involving in MSC-Exos to treat colitis. However, MSC-Exos may repair oxidative stress-damaged IECs and maintain the intestinal mucosal barrier. Additionally, MSC-Exos may alleviate oxidative stress injury through immune regulation. Therefore, it still needs further research whether MSC-Exos can reduce intestinal ROS production and regulate the balance between oxidation and antioxidation in the intestine.

*(4) Reduction of Intestinal Wall Permeability*. Rager et al. have found that the incidence of necrotizing enterocolitis (NEC), and intestinal wall permeability are decreased significantly in NEC model rats treated by intraperitoneal injection of BM MSCs or BM MSC-Exos [145]. In addition, McCulloh et al. have confirmed that MSC-Exos can reduce intestinal wall permeability, while the specific molecular mechanism remains unclear [146].

#### 3.4.2. Anticolonic fibrosis

Colonic fibrosis is a chronic complication of IBD. The incidence of fibrosis in patients with Crohn's disease (CD) is more than 30%, which quickly leads to intestinal stenosis and affects the prognosis of IBD patients [147, 148]. Pathological stimuli, such as chronic intestinal inflammation, oxidative stress injury, IEB damage, and repair, promote the occurrence of internal epithelial-mesenchymal transition (EMT), loss of polarity in epithelial cells, loss of intercellular connection, the transformation of morphology and function into EMT, and massive aggregation of extracellular matrix (ECM), which in turn leads to intestinal fibrosis [148–153]. During tissue repair or response, fibroblast activation is the key to fibrosis [154]. It has been found that the TGF-*β* signaling pathway activates fibroblasts and induces them to differentiate into myofibroblasts [155], which is the core link in the pathogenesis of intestinal fibrosis [156]. Yang et al. have found that BM-MSC-Exos can significantly reverse the EMT transformation of TGF-*β*1-treated intestinal epithelial cells-6 (IEC-6) cells through miR-200b, which significantly reverses intestinal fibrosis [157].

Studies have shown that MSCs from different sources mainly regulate EMT by activating TGF-*β* and Wnt signaling pathways to reduce ECM aggregation and produce antifibrotic effects [158]. Experiments have confirmed that MSCs from different sources can reduce the fibrosis of the heart, lung, liver, and kidney, while there is little research on intestinal fibrosis [159]. Choi et al. have found that umbilical cord/placenta MSC-ExoS downregulate TGF-*β*1-induced fibroblast activation by inhibiting the expression of Rho/MRTF/SRF, thereby suppressing intestinal fibrosis [160]. Duan et al. have also found that human placental MSC-Exos can reduce collagen production and promote collagen degradation by inhibiting the expression of TGF-*β*1 protein, thereby decreasing collagen deposition in the intestinal wall [161].

## 4. Therapeutic Potential of MSC-Exos in IBD

MSC-Exos have natural advantages in immune regulation and cell regeneration and repair. The application of MSC-Exos in disease models covers respiratory, cardiovascular, neurological, musculoskeletal, hepatic, gastrointestinal, cutaneous, and renal diseases. According to the Clinical Trials registered by the National Institutes of Health (NIH), the studies of MSC-Exos in clinical trials mainly include type I diabetes, cerebrovascular disease, autoimmune diseases, and macular hole [85, 162]. The application of ESCs-Exos in IBD starts late and is still in the animal experimentation stage.

### 4.1. Therapeutic Drugs

The MSC-Exo preparations can be administered through multiple routes, especially through the exploration of oral administration, which brings convenience to the treatment of IBD [163]. In animal experiments, it has been confirmed that MSC-Exos can relieve hematochezia, weight loss, and other colitis symptoms in animal models, improve histopathological scores, and enhance the survival rate of animals by oral administration, local enema, intraperitoneal injection, and intravenous injection [67, 92, 164]. The curative effect mainly focuses on short-term effects, such as inhibiting inflammatory reactions and repairing the intestinal barrier, to alleviate the symptoms of IBD rapidly. Long-term effects, such as the prevention of intestinal fibrosis and canceration of intestinal cells, are uncertain. MSC-Exos from different sources can contribute to the treatment of IBD through multiple therapeutic targets. Studies have shown that intravenous administration of hUC-MSC-Exos can target colonic tissue, reduce the severity of UC in mice, and improve survival. AD-MSC-Exos can reduce the typical symptoms of UC, decrease the histological score of DAI, and improve colonic ulceration. OE-MSC-Exos inhibit Th1/Th17 cells and enhance Treg cells to improve the severity of UC by inhibiting the inflammatory response [93]. In addition, local injection of MSCs has achieved good results in the treatment of IBD complicated with perianal fistula, and the clinical trial of MSC-Exo local injection in the treatment of IBD complicated with perianal fistula is in progress [165].

### 4.2. Drug Carrier

Conventional drug therapy for IBD relies on long-term, high-dose administration, and the accumulation of toxicity can lead to severe adverse reactions. Nanoparticle-based drug delivery systems (DDSs) can target intestinal inflammatory sites and enhance permeability and retention, reducing side effects and toxicity and improving therapeutic efficiency [163]. As a pure natural nanoparticle, MSC-Exo has targeting and low immunogenicity and can pass through different physiological barriers. After modification, MSC-Exos can effectively load different therapeutic agents (including protein, RNA, and chemotherapeutic drugs) and can be targeted and delivered to intestinal tissues to accumulate in inflammatory sites or cancer sites. Keener has found that doxorubicin (Dox) encapsulated in Exo reduces tumors more efficiently than the drug alone [166]. Han et al. have investigated human umbilical cord blood-derived MSC-Exos as a targeted delivery system for armodafinil (AMO) in colorectal cancer (CRC) therapy. Another study has used modified BM-MSC-Exos as a vehicle for Dox-targeted treatment of CRC in an animal model [167].

### 4.3. Diagnostic Markers

Exos from various types of cells can be detected in body fluids. Exos carry specific nucleic acids and proteins strictly screened by mother cells, which can reflect the state of cell metabolism and function. It has pronounced disease specificity. Therefore, Exos can be an ideal biomarker for disease diagnosis [168]. For example, the serum levels of Exo miR-21 can be used to differentiate esophageal squamous cell carcinoma from benign disease [169]. Compared with normal tissue Exos, lung cancer Exos show different levels of miRNAs (miR-378a, miR-379, miR-139-5p, and miR-200-5p), which can be used for screening and diagnosis of lung adenocarcinoma [170]. It is also worth looking forward to finding specific diagnostic biomarkers related to IBD in body fluid Exos.

## 5. Discussion and Perspectives

The pathogenesis of IBD is mainly intestinal cell damage mediated by abnormal immunity [171, 172]. According to the pathogenesis, both traditional drugs and biological agents can only meet the clinical needs of some IBD patients [4]. MSCs show strong immune regulation and tissue repair ability and can improve IBD from the root [19]. Animal experiments and clinical studies have confirmed that MSCs can improve IBD symptoms and reduce mortality by regulating intestinal immune abnormalities and maintaining the intestinal mucosal barrier [21, 22]. However, due to the potential immune rejection, abnormal differentiation, and tumorigenesis of cell therapy, the safety of the clinical application of MSCs has been questioned [57]. The efficacy and safety of MSCs from different sources and culture samples vary greatly [173]. Due to the lack of comparative studies, there is still a lack of scientific and rigorous explanations for the differences.

MSC-Exos are extracted from the culture medium of MSCs, which can achieve the therapeutic effect of MSCs and improve the safety and feasibility of the clinical application of MSCs [80]. The preclinical study of MSC-Exos in IBD has confirmed the efficacy and safety [36, 168]. However, the components of MSC-Exos are complex and heterogeneous, and the studies of molecular mechanisms are still very limited and face great challenges [78, 174]. The current research is limited to the possible effector molecules, most of which focus on the miRNA effect and have achieved certain results [36]. However, the source of preparation is mixed, the observation index is single, the determination of potency is complex, and the relationship between miRNA molecular content and effect cannot be unified [175]. Rich proteins still need to be further explored [78]. The research on neutral molecules and toxic molecules is far from enough.

The clinical application of MSC-Exos in IBD still faces many problems. The first is the heterogeneity of MSC-Exo preparations [37]. The source and culture conditions of MSCs will affect the biological characteristics of MSC-Exos and show differences in composition and biological functions [75]. At the technical level, the extraction of MSC-Exo preparations mainly relies on particle size and density for separation and purification and lacks highly sensitive and specific molecular markers. It is challenging to obtain relatively pure MSC-Exo preparations due to the mixing of various sEV subtypes or biological macromolecules of similar size. The diversity of extraction methods leads to the different purities of MSC-Exo preparations, affecting the rigor of the study and the comparison and repeatability between studies [83]. The identification methods of MSC-Exo preparation are diverse, and the technology is not mature enough. Therefore, there are still errors in determining the purity and composition of MSC-Exo preparations [84]. Whether it is the physical performance of MSC-Exos or the production technology of MSC-Exos, the heterogeneity generated in each link can be better applied in clinical practice by comparative research, optimization and improvement, and unified scheme and technology. The second is MSC-Exo potency determination. Compared with the determination of purity and composition of MSC-Exos, the determination of activity and potency of MSC-Exos is more challenging [83]. The content and activity of effector molecules in MSC-Exos are measured to predict their therapeutic effect in IBD. However, the molecular components of MSC-Exos are complex, and the effector molecules and specific effects of MSC-Exos in IBD still need to be further studied. At present, its efficacy can only be evaluated by detecting the most relevant biological activity that MSC-Exos can expect, which is difficult to reflect the molecular MoA of MSC-Exo preparations in IBD [84]. Moreover, there is no consensus on the infusion route and dosage of MSC-Exo preparations in IBD patients [168]. To increase targeting and reduce side effects, comparative research has been carried out to optimize the infusion scheme and to promote the clinical transformation of MSC-Exos in IBD.

MSCs and MSC-Exos have been investigated in clinical research or applied in many fields of clinical medicine with their immune regulation, tissue regeneration, and repair abilities. The application of MSC-Exo preparations will have a bright future for the immune injury of the IBD digestive tract. Currently, the local application of MSC-Exo preparations has obtained positive clinical efficacy in perianal fistula Crohn's disease (PFCD) [176]. With the increasingly mature technology of cell culture and exocrine extraction, the molecular mechanism underlying the effects of Exos is continuously studied in depth, which can provide more optimized MSC-Exo preparations, gradually expand the scope of application in IBD, and become an effective means of IBD treatment.

## Figures and Tables

**Table 1:** Therapeutic application of MSC-Exo in IBD treatment.

Exosomes sources	Effector molecule	Mechanism	Effect	Ref.
hUC-MSCs	TSG-6	Repair TJ, increasing the expression of TJ proteins. Modulated immune response of Th2 and Th17 cells.	Restoring mucosal barrier repair and intestinal immune homeostasis.	[23]
hBM-MSCs	MT-2	Polarizing M2b macrophages.	Reduce mucosal inflammation.	[96]
hUC-MSCs	miRNA-326	NF-*κ*B signaling pathway, neddylation-related enzymes.	Inhibiting the neddylation and alleviating colitis.	[177]
hUC-MSCs	miRNA-378a-5p	NLRP3 axis; IL-1*β*; IL-18; and Caspase-1.	Attenuating colitis by regulating macrophage pyroptosis.	[93]
hUC-MSCs	miRNA-378a-5p	NLRP3 axis	Regulating macrophage pyroptosis and protecting against DSS-induced colitis.	[178]
AD-MSCs	miRNA-132	Smad-7 and TGF-*β*/Smad signaling.	Promoting VEGF-C-dependent lymphangiogenesis.	[179]
hUC-MSCs	miRNA-146a	SUMO1 axis.	Preventing colitis.	[177]
hUC-MSCs	lnc78583-miRNA-3202	HOXB13 axis.	Relieve inflammatory bowel disease.	[180]
BM-MSCs	MiRNA-200b	HMGB3 axis.	Attenuate the inflammatory injury of IECs.	[181]
BM-MSCs	MiR-125a miR-125b	Stat3 axis; inhibit Th17 cells differentiation.	Attenuate DSS-induced colitis.	[182]
AD-MSCs	N/A	Immunomodulatory effects; regulate Treg population.	Alleviate inflammation in DSS-induced acute colitis	[114]
Olfactory Ecto-MSCs	N/A	Regulates subpopulations and differentiation of Th1/Th17.	Alleviate the disease severity of IBD.	[113]
hUC-MSCs	N/A	Down-regulation the expression of iNOS and IL-7.	Relieve inflammatory responses, and attenuates IBD.	[93]
hUC-MSCs	N/A	Regulate the modification of ubiquitination.	Attenuate the severity of colitis.	[67]
BM-MSCs	N/A	Attenuate colon inflammation, oxidative stress, and apoptosis.	Relieve the severity of IBD.	[129]
AD-MSCs	N/A	Immunity, apoptosis, and inflammation pathway.	Reduce intestinal epithelial damage.	[183]

AD-MSCS: adipose mesenchymal stem cells; DSS: dextran sulfate sodium; Exo: exosomes; hBM-MSCs: human bone marrow mesenchymal stem cells; HMGB3: high-mobility group box 3 protein; HOXB13: high-mobility group box 13 protein; hUC-MSCs: human umbilical cord mesenchymal stem cells; IBD: inflammatory bowel diseases; IECs: induced embryonic stem cells; IL-18: interleukin-18; IL-1*β*: interleukin-1*β*; IL-7: interleukin-7; iNOS: inducible nitric oxide synthase; MT-2: metallothi-onein-2; N/A: not Available; NF-*κ*B: nuclear factor kappa-B; NLRP3: NOD-like receptor thermal protein domain associated protein 3; Olfactory Ecto-MSCs: olfactory ecto mesenchymal stem cells; Stat3: signal transducer and activator of transcription 3; SUMO1: small ubiquitin-like modifier 1; TGF-*β*: transforming growth factor-*β*; Th17: T helper 17 cells; Th2: T helper 2 cells; TJ: tight junctions; TSG-6: tumor necrosis factor-*α* stimulated gene 6; VEGF: vascular endothelial growth factor.

## Abbreviations

MSCs: Mesenchymal stem cells
MSC-Exo: Mesenchymal stem cell-derived exosomes
IBD: Inflammatory bowel disease
TNF-*α*: Tumor necrosis factor-*α*
EVs: Extracellular vesicles
ESCRT: Endosomal sorting complex required for transport
TRAF-6: TNF receptor related factor 6
IRAK-1: IL-1 receptor related kinase 1
MT-2: Metallothionein-2
TLR: Toll-like receptors
CCR2: CC Chemokine Receptor 2
DCs: Dendritic cells
hUC-MSC-Exo: Human umbilical cord MSC-Exo
MLN: Mesenteric lymph nodes
AD-MSC-Exo: Adipose-MSC-Exo
DSS: Dextran sulfate sodium
ROS: Reactive oxygen species
IECs: Intestinal epithelial cells
NEC: Necrotizing enterocolitis
ADSCs-Exo: Adipocyte derived stem cells-Exo
VEGF-C: Vascular endothelial growth factor C
LECs: Lymphatic endothelial cells
CD: Crohn disease
EMT: Epithelial mesenchymal transition
ECM: Extracellular matrix
TGF-*β*: Transforming growth factor-*β*
IEC-6: Intestinal epithelial cells-6.
